# Sequential Processing Enables 17% All-Polymer Solar Cells via Non-Halogen Organic Solvent

**DOI:** 10.3390/molecules27175739

**Published:** 2022-09-05

**Authors:** Chaoyue Zhao, Lihong Wang, Guoping Zhang, Yajie Wang, Ruiyu Hu, Hui Huang, Mingxia Qiu, Shunpu Li, Guangye Zhang

**Affiliations:** College of New Materials and New Energies, Shenzhen Technology University, Shenzhen 518118, China

**Keywords:** all-polymer solar cells, sequential processing, polymerized small molecule acceptors, non-halogen solvent, power conversion efficiency

## Abstract

All-polymer solar cells (All-PSCs), whose electron donor and acceptors are both polymeric materials, have attracted great research attention in the past few years. However, most all-PSC devices with top-of-the-line efficiencies are processed from chloroform. In this work, we apply the sequential processing (SqP) method to fabricate All-PSCs from an aromatic hydrocarbon solvent, toluene, and obtain efficiencies up to 17.0%. By conducting a series of characterizations on our films and devices, we demonstrate that the preparation of SqP devices using toluene can effectively reduce carrier recombination, enhance carrier mobility and promote the fill factor of the device.

## 1. Introduction

With the increasing focus on new energy technology, organic solar cells (OSCs) have become one of the hottest research directions in the field of thin film photovoltaics [1]. In addition to their extremely thin active layer, e.g., ~100 nm, OSCs have many features that traditional photovoltaics do not have, such as flexibility [2,3], semitransparency [4], low-cost printing production [5], etc., and are considered one of the most promising next-generation PV technologies for commercialization [6,7]. In the near future, the market entry of OSCs will strongly depend on the improvement of their photovoltaic conversion efficiency (PCE) and stability [8,9]. In the past few years, the most successful strategy to improve the PCE has involved alternating the materials used in the active layer. Specifically, by replacing the conventional fullerene acceptors with non-fullerene small molecules or polymers, the PCE of OSCs have increased dramatically within a rather short time frame [10,11,12,13,14]. Recently, Hui Huang et al. fabricated 19% OSCs devices by using a polymer donor and a non-fullerene small molecule acceptor to construct the active layer [15].

When the electron donor is a polymer and the non-fullerene acceptor is also a polymer, the solar cell is typically referred to as an all-polymer solar cell (All-PSC) [16,17]. Due to their similar advantages to small molecule acceptor-based OSCs, All-PSCs possess better mechanical properties, such as higher tensile and flexural toughness, and potentially higher thermal stability, which give them better prospects for mass production [18,19,20]. Recently, the strategy of polymerized small molecule acceptors (PSMAs) has boosted the efficiency of All-PSCs to over 17% [21,22]. However, most of the high PCEs for these all-PSCs have been based on active layers that were processed from either chloroform (CF) or a CF-dominant solvent mixture [20,23,24]. The treatment of the active layer with this low boiling point, carcinogenic solvent inhibits mass-production and can be only used to evaluate the potential of a material at a laboratory scale. In addition, most of the previous studies are based on the one-step blend casting (BC) method for active layer film formation, which is not conducive to the optimization of the vertical distribution of materials in the active layer, which also limits device performance [25,26,27,28,29].

In this work, we used toluene (Tol), an aromatic hydrocarbon solvent which is less toxic and has a boiling point as high as the solvent, to make all-PSC devices. In order to optimize both the bulk morphology and vertical phase segregation, we use the sequential processing (SqP) technique to prepare the active layer instead of the BC method. SqP, also called layer-by-layer (LBL), quasi-bilayer, pseudo-bilayer, etc. in the literature, has been shown to be an effective method to prepare the active layer of organic solar cells in both small and large areas with enhanced performance, batch-to-batch consistency and even stability [30,31]. A detailed description of the SqP method and its advantages can be found in our previous study and in the literature [25,26,32]. In the BC method, the efficiency of devices prepared with Tol as solvent is usually inferior to that of CF solvent. Herein, by sequentially processing the donor PM6 and a PSMA named PY-V-*γ*, we, for the first time, obtained All-PSCs devices with an efficiency of 17.0% using toluene as the main solvent. Through film-depth-dependent light absorption spectra (FLAS) experiments, we found that vertical phase segregation can be significantly improved by the SqP method, which leads to a large increase in the FF and, thus, the efficiency of the device. In order to further investigate the device physics, we carried out transient photovoltage (TPV) and transient photocurrent (TPC), Fourier-transform photocurrent spectroscopy external quantum efficiency (FTPS-EQE), light intensity dependent *J-V* measurement, etc., which revealed that the SqP method can effectively reduce charge recombination, increase and balance charge transport and enhance the charge carrier lifetime.

## 2. Experimental

### 2.1. Materials

PM6 was purchased from Solarmer Material Inc. (Beijing, China). PY-V-*γ* and PNDIT-F3N were purchased from eFlexPV Limited (Guangdong, China). PEDOT:PSS (Clevios P VP 4083) was purchased from Heraeus Inc. (Hanau, Germany). All the other reagents and chemicals were purchased from Sigma Aldrich or Aladdin and used as received.

### 2.2. Experimental Equipment and Facilities

**Device fabrication.** Solar cells were fabricated in a conventional device configuration of ITO/PEDOT:PSS/active layer/PNDIT-F3N/Ag. The ITO substrates were cleaned by detergent and then sonicated with deionized water, acetone and isopropanol, and dried overnight in an oven. The glass substrates were treated with UV-Ozone for 30 min before PEDOT:PSS was spin-casted on top at 5000 rpm for 30 s, and then annealed at 150 °C on a hotplate for 10 min in air.

BC and SqP devices:

(1) For the blend-casting (BC) devices, namely, PM6:PY-V-*γ* (BC), the PM6: PY-V-*γ* blend (1:1.2 weight ratio) was dissolved in toluene (the concentration of donor was 7 mg mL^−1^ for all blends), with 1-chloronaphthalene (1% vol) as an additive and stirred overnight in a nitrogen-filled glove box. The 95 °C toluene blend solution was spin-casted at 2500 rpm for 30 s onto the PEDOT:PSS films followed by a thermal annealing of 95 °C for 5 min.
(2) For the sequentially processed (SqP) device, namely, PM6/PY-V-*γ* (SqP), PM6 was dissolved in toluene (the concentration of donor was 8 mg mL^−1^); PY-V-*γ* was also dissolved in toluene (the concentration of donor was 12 mg mL^−1^) but with 1-chloronaphthalene (2% vol) as an additive. Both solutions were stirred overnight in a nitrogen-filled glove box. The donor solution was spin-casted at 4000 rpm for 30 s onto the PEDOT:PSS films, then the acceptor solution was spin-casted at 4000 rpm for 30 s onto the donor films followed by a thermal annealing of 95 °C for 5 min.

For all types of devices, a methanol with 0.5% vol acetic acid blend solution of PNDIT-F3N at a concentration of 0.5 mg mL^−1^ was spin-coated onto the active layer at 2000 rpm for 30 s. Around 100 nm of Ag was evaporated under 4 × 10^−4^ Pa through a shadow mask. Then, the encapsulation was carried out.

**Device characterization.** The details of *J-V* testing, and EQE measurements are shown in the SI.

### 2.3. Analysis and Characterization

**SCLC Measurements:** The electron- and hole-mobilities were evaluated using the space-charge limited current (SCLC) method. The device architecture of the electron-only devices was ITO/PNDIT-F3N/active layer/PNDIT-F3N/Ag and that of the hole-only devices was ITO/PEDOT:PSS/active layer/ Au. The charge carrier mobilities were determined by fitting the dark current into the model of a single carrier SCLC according to the equation: *J* = 9*ε*_0_*ε*_r_*μV*^2^/8*d*^3^, where *J* is the current density, *d* is the film thickness of the active layer, *μ* is the charge carrier mobility, *ε*_r_ is the relative dielectric constant of the transport medium, and *ε*_0_ is the permittivity of free space. The *V* used in the equation is defined by: *V* = *V*_app_ –*V*_bi_, where *V*_app_ is the applied voltage, *V*_bi_ is the built-in voltage. The carrier mobilities were calculated from the slope of the *J*~*V*^2^ curves.

**FTPS-EQE:** FTPS-EQE measurements were conducted on the same devices used in *J-V* measurements using an integrated system (PECT-600) purchased from Enli Technology Co., LTD (Taiwan, China). The range of measurements was 600–1500 nm. The signal due to noise was cut out for all devices below 0.83–2.07 eV. 

**Film-depth-dependent light absorption spectroscopy (FLAS) and composition distribution:** Film-depth-dependent light absorption spectra were acquired by an in situ spectrometer (PU100, Shaanxi Puguang Weishi Co. Ltd.) (Shaanxi, China) equipped with a soft plasma-ion source. The power-supply for generating the soft ionic source was 100 W with an input oxygen pressure ~10 Pa. The film surface was incrementally etched by the soft ion source, without damage to the materials underneath the surface, which was in situ monitored by a spectrometer. From the evolution of the spectra and the Beer–Lambert’s Law, film-depth-dependent absorption spectra were extracted. 

The composition distribution along the film-depth direction was obtained from the film-depth-dependent spectra by fitting the sub-layer absorption using the absorption of the pure components. The exciton generation contour is numerically simulated upon inputting sub-layer absorption spectra into a modified optical transfer-matrix approach. The detailed experimental and numerical method are available elsewhere [33,34].

## 3. Results and Discussion

**Figure 1:**
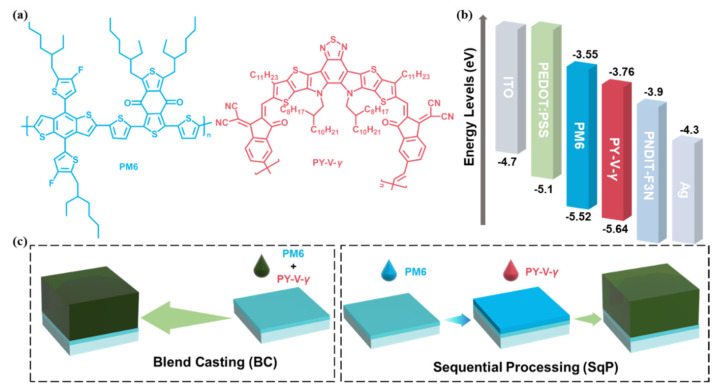
(**a**) Chemical structures of PM6 and PY-V-*γ*. (**b**) Energy level diagram. The bottom and top of the bar represent HOMO and LUMO, respectively. (**c**) Schematic diagram of blend-casting and sequential processing.

The chemical structures of the polymers, the energy levels and device fabrication schematic are shown in Figure 1, where different active layer deposition processes, blend-casting (BC) and sequential processing (SqP) are depicted in an abridged general view in Figure 1c. The chemical structural formulas of PM6 and PY-V-*γ* are shown in Figure 1a. The highest occupied molecular orbital (HOMO) and lowest unoccupied molecular orbital (LUMO) energy levels of the materials are literature values [35]. As Figure 1b shows, the energy level matching of each layer is favorable, and such a combination can maximize the efficiency and reduce the energy loss. The two devices with PM6 and PY-V-*γ* as the active layer materials were fabricated, named PM6:PY-V-*γ* (BC) and PM6/PY-V-*γ* (SqP). The final overall structure of the device is ITO/PEDOT:PSS/active layer/PNDIT-F3N/Ag, where PEDOT:PSS and PNDIT-F3N are the hole and electron transport layer, respectively. 

**Figure 2:**
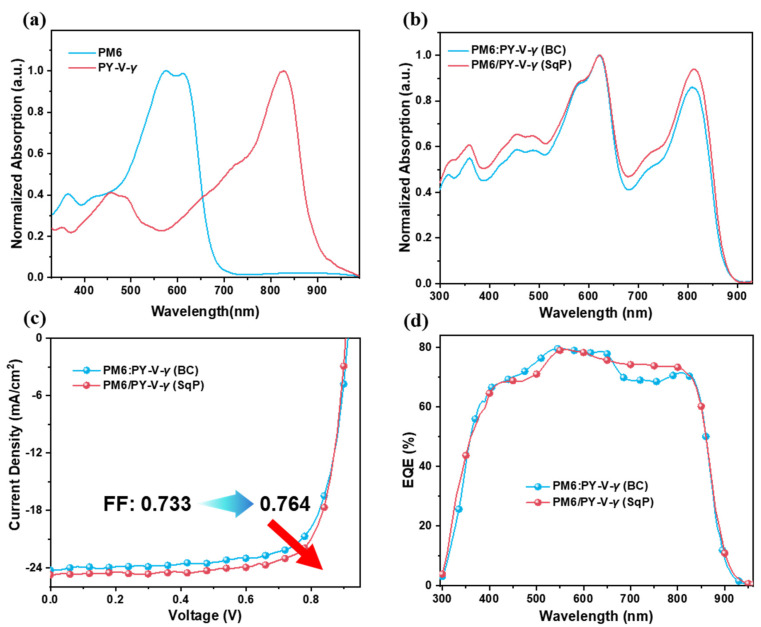
(**a**) UV-Vis absorption spectra of pure PM6 and pure PY-V-*γ*. (**b**) UV-Vis absorption spectra of blend films of PM6 and PY-V-*γ* made from BC or SqP. (**c**) External quantum efficiency (EQE) spectra of the BC and SqP devices. (**d**) Current density-voltage (*J*-*V*) curves.

Figure 2a shows the UV-Vis absorption spectra of the thin films of the pure materials spin-coated from toluene. The normalized absorption spectra of the blend film are shown in Figure 2b, where we find that the UV-Vis absorption spectra are mainly divided into three parts. The peak in the region of 300–400 nm has a contribution from both PM6 and PY-V-*γ*. Another peak at 400 nm–700 nm is from the absorption of PM6, while that in the range of 700–900 nm is the absorption of PY-V-*γ*. The blend covers the majority of the solar spectrum thanks to the complementary absorption between the donor and the acceptor. The current density and voltage (*J*-*V*) characteristics of these two devices are shown in Figure 2c. The calculated photovoltaic parameters are summarized in Table 1. The device with the BC method exhibits a PCE of 16.3% with a *V*_OC_ of 0.913 V, a *J*_SC_ of 24.28 mA cm^−2^, and an FF of 0.733. Then, the device fabricated with SqP method shows an elevated FF (0.764), which resulted in the highest PCE of 17.0%. These results demonstrate that the voltage and current for the devices based on BC and SqP do not differ significantly, while the main difference comes from the FF. The significantly enhanced FF from 0.733 to 0.764 leads to the efficiency improvement from 16.3% to 17.0%. More spin-coating conditions were tested, and the corresponding device *J-V* characteristics can be found in the SI. Figure 2d shows the EQE of each device. We found that the slight variation in the *J*_SC_ mainly arises from the EQEs in the range of ~680–800 nm, which is consistent with our previous observation [36]. Combined with Table S1, the *J*_SC_s of the device is consistent with the integrated value from the EQE curves, with an error within 5%. 

**Figure 3:**
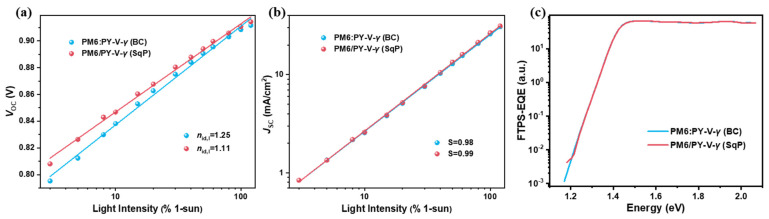
(**a**) *J*_SC_ versus light intensity; (**b**) *V*_OC_ versus light intensity; (**c**) FTPS-EQE spectra.

**Figure 4:**
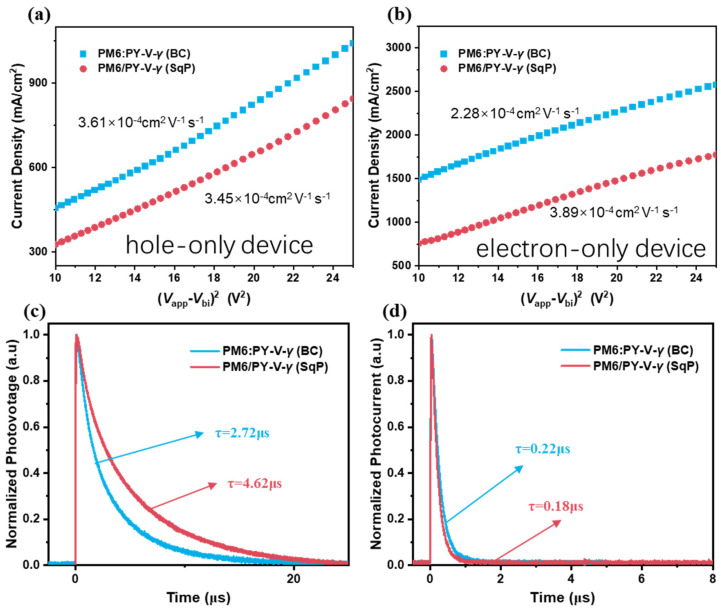
*J*-*V*^2^ curves of hole-only devices (**a**) and electron-only devices (**b**) for SCLC mobility evaluation. TPV (**c**) and TPC (**d**) decays of the BC and SqP devices.

To better understand the device performance (Figure 3 and Table 1), light intensity studies were first performed to analyze the charge recombination. First, the change in *J*_SC_ with respect to light intensity is plotted in Figure 4a on a logarithmic scale. The slope (*S*) obtained from linear fittings is listed in Table 1, which has been adopted in the field to indicate bimolecular recombination. The *S* values for the BC and SqP devices are 0.98 and 0.99, respectively, indicating rather similar bimolecular recombination. Next, by fitting the *V*_OC_ versus ln(*I*) result, ideality factors (*n*_id_) can be calculated, which has been shown to relate to trap-assisted recombination based on the diode theory. A higher *n*_id_ indicates more trap-assisted recombination [37]. Figure 3b shows the dependence of the *V*_OC_s on light intensity for the SqP and BC devices, and Table 1 shows the fitting result. The ideality factors (*n*_id,l_) under different light intensities can by calculated by ${𝓃}_{\mathrm{id},l}=\frac{q}{kT}\frac{\partial V_{\mathrm{oc}}}{\partial \ln\left(I\right)}\;$, where *I* is the light intensity, *q* the elementary charge, *k* the Boltzmann constant, and *T* the Kelvin temperature. Compared to the BC device which shows a *n*_id,l_ of 1.25, the *n*_id,l_ of SqP devices is 1.11, implying less trap-assisted recombination and the carrier recombination type is mainly bimolecular (*S* close to 1). To further verify the ideality factor between SqP and BC, we fit the exponential region of the dark *J-V* curves, where the dark ideality factor (*n*_id,d_) can be calculated by ${𝓃}_{\mathrm{id},d}=\frac{q}{kT}\frac{\partial V}{\partial J}$, where *q*, *k*, *T*, *V*, and *J* are the fundamental charge, Boltzmann constant, bias voltage and dark current density, respectively. The dark *J-V* curves are plotted in Figure S1. Table S1 shows the *n*_id,d_ calculated using the slope of the linear region (see Figure S1). In addition, we also carried out Fourier-transform photocurrent spectroscopy external quantum efficiency (FTPS-EQE), which can detect the weak current in the low energy (near infrared) region and, thus, allows us to probe the states inside the bandgap. As shown in Figure 3c, the BC device shows a longer tail in the low-energy region than the SqP device, indicating that the BC device may have more defect states within the bandgap.

To assess the charge transport properties of different devices, we fabricated hole-only and electron only devices and plotted the *J−V*^2^ curves of them. By fitting the result to the space charge limited current (SCLC) model, hole-mobility (*μ*_h_) and electron-mobility (*μ*_e_) can be calculated. As shown in Table 1 and Figure 4a,b, the hole mobilities between the SqP (3.45 × 10^−4^ cm^2^ V^−1^ s^−1^) and BC devices (3.61 × 10^−4^ cm^2^ V^−1^ s^−1^) are similar, but electron mobilities increase from 2.28 × 10^−4^ cm^2^ V^−1^ s^−1^ (BC) to 2.28 × 10^−4^ cm^2^ V^−1^ s^−1^ (SqP). This increase makes the SqP device more balanced in charge transport, consistent with its high FF. Next, to study the carrier lifetime and carrier extraction time, we carried out transient photovoltage (TPV) and transient photocurrent (TPC) experiments. The normalized TPV and TPC curves are shown in Figure 4c,d. The carrier lifetime (τ) of the SqP device is 4.62 μs, which exceeds that of the BC device (τ = 2.72 μs). From the TPC, the carriers of the SqP device are extracted faster (0.18 μs) than the BC device (0.22 μs). The trend of the TPV and TPC results are consistent with the result we obtained in other analyses, e.g., the trend in trap-assisted recombination, the density of defect states indicated by FTPS-EQE and the trend in FF of the devices.

**Figure 5:**
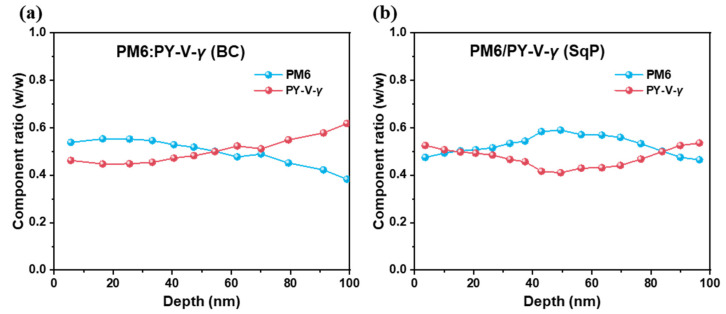
The composition ratio across the vertical direction of the active layer for BC PM6:PY-V-*γ* (**a**) and SqP PM6/PY-V-*γ* (**b**).

**Figure 6:**
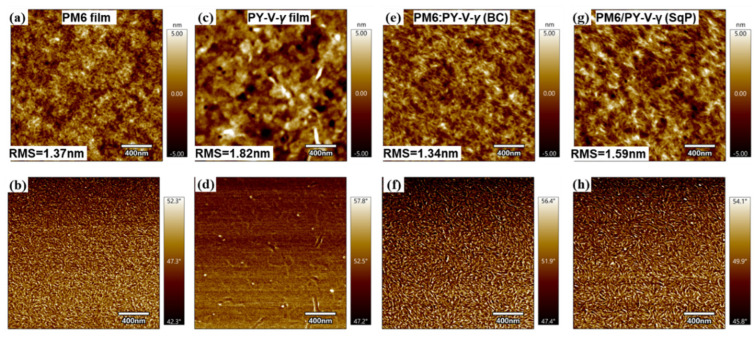
AFM height (top) and phase images (bottom) of (**a**,**b**) PM6 film, (**c**,**d**) PY-V-*γ* film, (**e**,**f**) PM6:PY-V-*γ* (BC), (**g**,**h**) PM6/PY-V-*γ* (SqP).

After analyzing the device physics, we turn to study the morphology. One of the initial motivations of using SqP was that it could induce better vertical phase separation. To probe it, we performed film-depth-dependent light absorption spectroscopy (FLAS). This technique utilizes low-pressure plasma to incrementally etch the film from the top surface (electron transport layer/active layer interface) to the bottom of the active layer (hole transport layer interface). By measuring the underlayer absorption in situ, FLAS can provide the absorption of the sub-layers. Fitting the sub-layer absorption with the pure component absorption, the composition distribution for each sublayer can be obtained. Figure 5, Figures S2 and S3 are the result for the BC and SqP films from top (0 nm) to bottom (100 nm). By comparing Figure 5a,b, we find that the the acceptor, PY-V-*γ*, tends to segregate toward the bottom (anode) in the BC device. Such more-acceptor-less-donor distribution in the bottom part of the device is unfavorable, which could induce more surface recombination and block holes from collecting by the anode. In contrast, SqP film shows a much-improved vertical phase segregation, where the large fraction of PY-V-*γ* is much reduced near the bottom and that the fraction of PY-V-*γ* at the top surface is higher than that in the BC method. This improved the vertical phase separation, can reduce surface recombination and thus be another factor for the high FF of the SqP device. After testing FLAS for different active layer thicknesses, as shown in Figure S5, we also confirmed that the vertical phase morphology is not consistent for different device thicknesses. On the other hand, it also shows that the SqP method is more sensitive to the modulation of the vertical morphology. Using the transfer matrix model and the optical constants of the different layers including the sub-layers of the active layer (see reference [38] for computational details), in Figure 6, we show the atomic force microscopy result on the SqP and BC films. The AFM images show that there exist more PY-V-*γ* and less PM6 at the top surface of the SqP film than the BC film, which is consistent with the FLAS result, confirming the better vertical phase separation for the SqP film. Furthermore, a root mean square (RMS) roughness of 1.34 nm of the BC device is observed, close to the RMS roughness of pure PM6. In contrast, the RMS roughness of 1.59 nm for SqP device and the RMS = 1.82 nm for pure PY-V-*γ* indicate that there may be more acceptor PY-V-*γ* on the upper surface of the SqP device, further confirming the visual results.

## 4. Conclusions

In conclusion, in the all-polymer material system of donor PM6 and acceptor PY-V-*γ*, the SqP method allowed us to process the active layer from toluene, a greener and more industry-compatible solvent than chloroform, and obtain an excellent PCE of 17.0%. In comparison, the toluene processing provides an efficiency of 16.3% for the device processed from the conventional BC method. The characterization showed that SqP method effectively reduced the carrier recombination, balanced charge transport, improved the vertical phase segregation and thus improved the FF (0.764) and PCE of the device. At the same time, the SqP method is able to separately modulate the donor and acceptor layers, making it easier to optimize the morphology of the active layer, resulting in a significant increase in electron mobility. These advantages significantly promote the significance of the SqP method in all-PSCs and the use of the SqP method in toluene solvent has far-reaching implications for future large-area fabrication of organic solar cells.

## Figures and Tables

**Table 1:** Summary of photovoltaic parameters for PM6 and PY-V-*γ* based all-PCSs processed from different methods, measured under AM 1.5 G illumination at 100 mW cm^−2^.

Active Layer	*V*_OC_ [V]	*J*_SC_ [mA/cm^2^]	FF	PCE ^(a)^ [%]	*S* ^(b)^	*n*_id,l_^(c)^	*μ*_h_ [cm^2^ V^−1^ s^−1^]	*μ*_e_ [cm^2^ V^−1^ s^−1^]
BC	0.909 ± 0.003 (0.913)	24.0 ± 0.3 (24.3)	0.737 ± 0.006 (0.733)	16.1 ± 0.1 (16.3)	0.983	1.245	3.61 × 10^−4^	2.28 × 10^−4^
SqP	0.906 ± 0.001 (0.906)	24.3 ± 0.2 (24.5)	0.765 ± 0.005 (0.764)	16.8 ± 0.1 (17.0)	0.991	1.114	3.45 × 10^−4^	3.89 × 10^−4^

^(a)^ The standard deviations are based on measurements of over at least ten independent devices; ^(b)^ The slope from the linear fit of *J*_SC_ versus log*I*; ^(c)^ Ideality factors obtained from analyzing *V*_OC_-light intensity data.

## Data Availability

Not applicable.

## Supplementary Materials

The following supporting information can be downloaded at: https://www.mdpi.com/article/10.3390/molecules27175739/s1; Figure S1. dark *J*–*V* curves of BC and SqP; Figure S2. (a,b) Film-depth-dependent light absorption spectra, c)-d) absorption of the sub-layer inside the active layer calculated from FLAS spectra. The spectra are vertically shifted and rescaled for clarity; Figure S3. (a,b) Integrated generation rate in the vertical direction of the film; c-d) Exciton generation map across the vertical direction of the active layer film as a function of wavelength; Figure S4. The stability of BC and SqP device; Figure S5. Comparison of FLAS tests with different spin speeds to obtain different active layer thicknesses, based on SqP devices: (a,f,k) Film-depth-dependent light absorption spectra; (b,g,l) absorption of the sub-layer inside the active layer calculated from FLAS spectra; The spectra are vertically shifted and rescaled for clarity; (c,h,m) The composition ratio across the vertical direction of the active layer; (d,i,n) Integrated generation rate in the vertical direction of the film; (e,j,o) Exciton generation map across the vertical direction of the active layer film as a function of wavelength; Table S1. EQE and *n*_id,d_ of BC and SqP.
