# Supplementary material for: Sequential Processing Enables 17% All-Polymer Solar Cells via Non-Halogen Organic Solvent
Source: Molecules. 2022 Sep 5;27(17):5739. doi: 10.3390/molecules27175739 (PMC9458225; doi:10.3390/molecules27175739)
Supplement: Supplementary file 1 [file molecules-27-05739-s001.zip › molecules-1888090-supplementary.pdf]

molecules

Supporting Information

Sequential Processing Enables 17% All-Polymer Solar Cells via Non-Halogen Organic Solvent

Chaoyue Zhao [1]†, Lihong Wang[1]†, Guoping Zhang [1], Yajie Wang[1], Ruiyu Hu [1], Hui Huang[1], Mingxia Qiu[1], Shunpu Li[1] and Guangye Zhang[1*]

[1] College of New Materials and New Energies, Shenzhen Technology University, Shenzhen 518118, China
* Correspondence: zhangguangye@sztu.edu.cn
† These authors contributed equally to this work

Device characterization.

The current density-voltage (*J-V*) curves of all encapsulated devices were measured using a Keithley 2400 Source Meter under AM 1.5G (100 mW cm^{-2}) using an Enlitech solar simulator. The light intensity was calibrated using a standard Si diode with KG5 filter to bring spectral mismatch to unity. Optical microscope (Olympus BX51) was used to define the device area (8.5 mm^2). EQEs were measured using an Enlitech QE-S EQE system equipped with a standard Si diode. Monochromatic light was generated from a Enlitech 300W lamp source.

Table S1. EQE and $n_{id,d}$ of BC and SqP.

active layer	J_{EQE} [mA/cm^2]	$n_{id,d}$ [a)]
BC	23.12	1.32
SqP	23.36	1.33

[a)] Ideality factors obtained from fitting dark *J–V* curves.

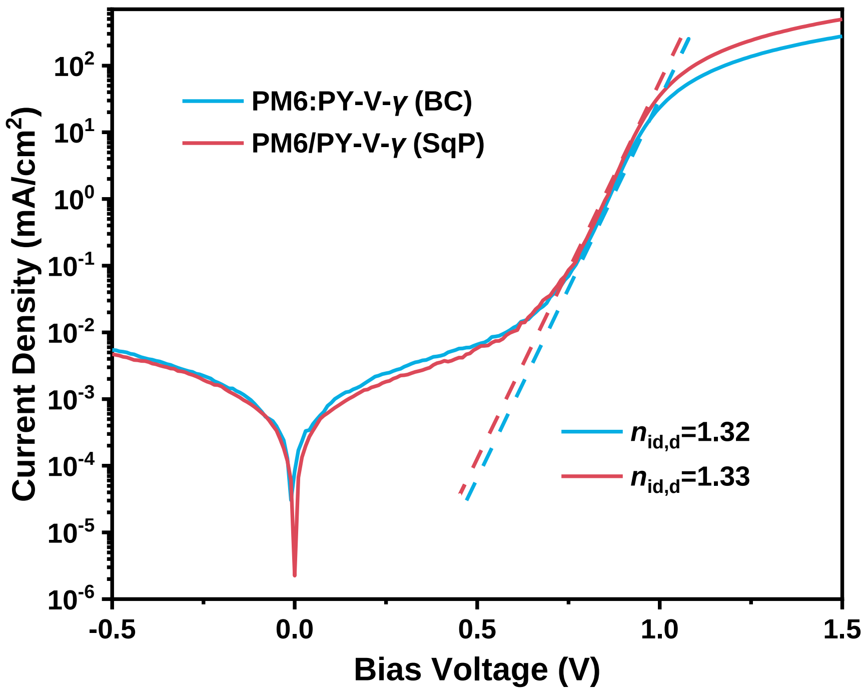

Figure S1. dark *J–V* curves of BC and SqP.

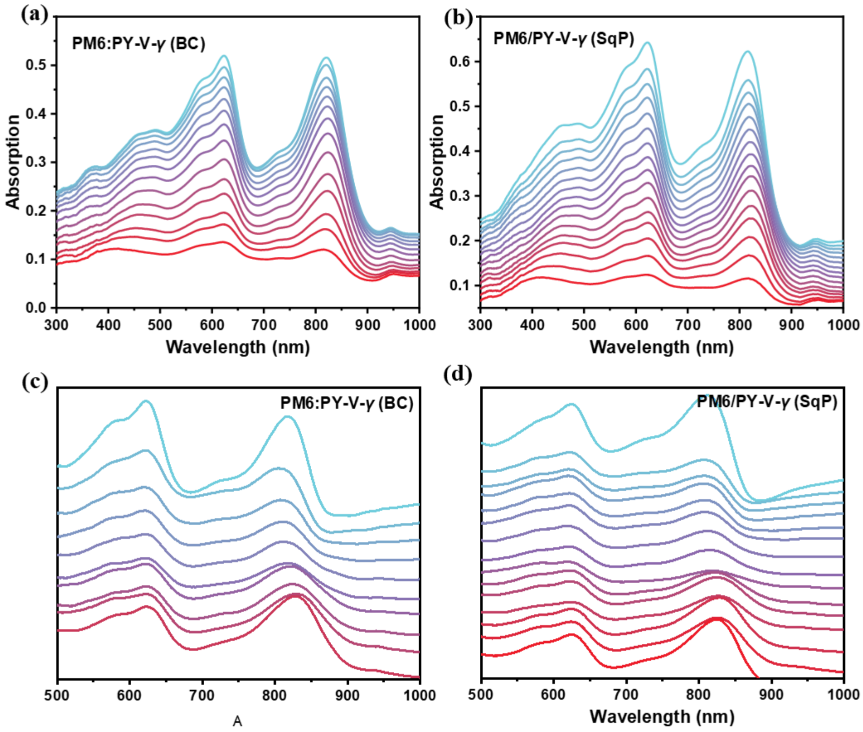

Figure S2. a) and b) Film-depth-dependent light absorption spectra, c)-d) absorption of the sub-layer inside the active layer calculated from FLAS spectra. The spectra are vertically shifted and rescaled for clarity.

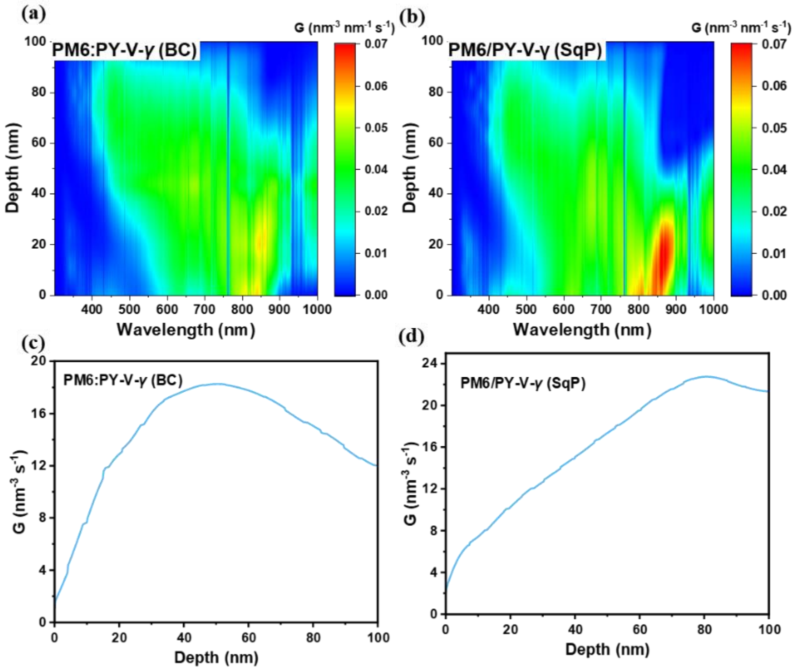

Figure S3. a-b) Integrated generation rate in the vertical direction of the film; c-d) Exciton generation map across the vertical direction of the active layer film as a function of wavelength.

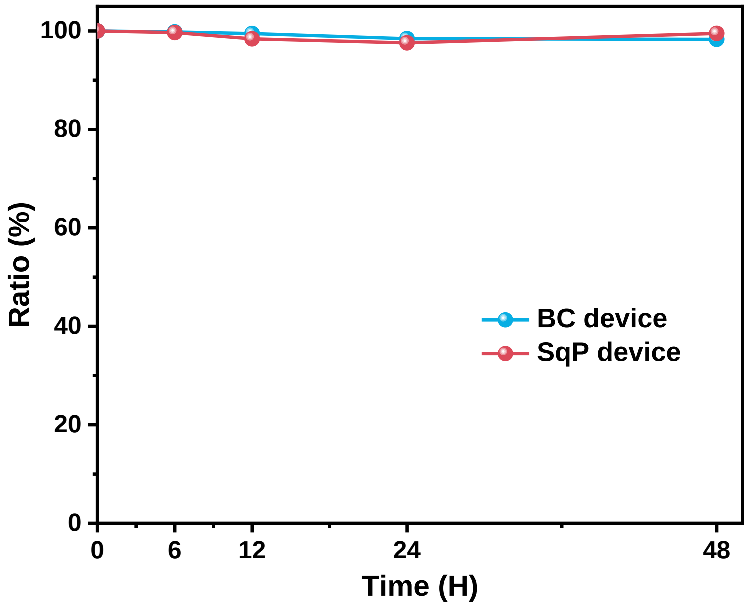

Figure S4. The stability of BC and SqP device.

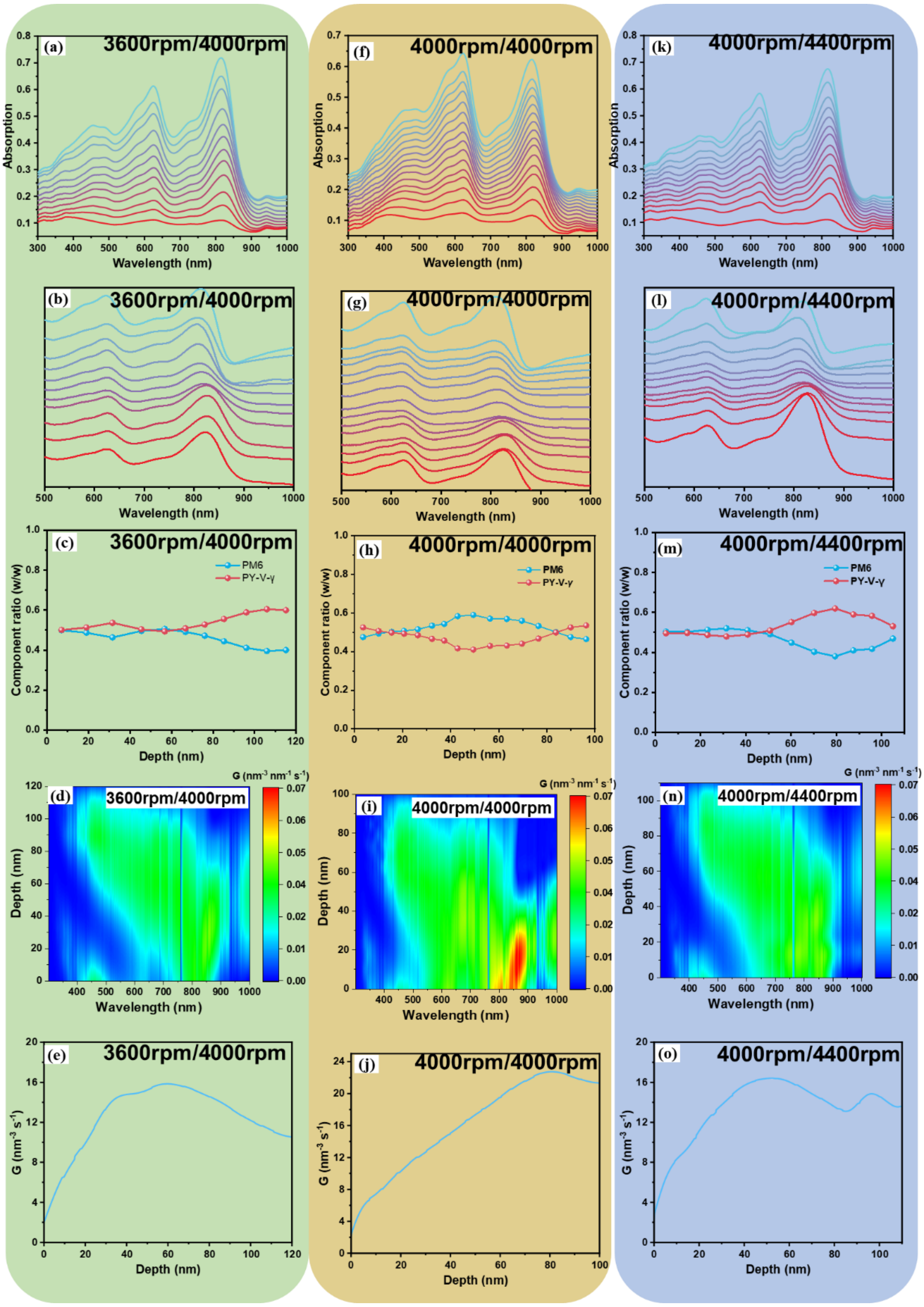

Figure S5. Comparison of FLAS tests with different spin speeds to obtain different active layer thicknesses, based on SqP devices: (a, f, k) Film-depth-dependent light absorption spectra; (b, g, l) absorption of the sub-layer inside the active layer calculated from FLAS spectra; The spectra are vertically shifted and rescaled for clarity; (c, h, m) The composition ratio across the vertical direction of the active layer; (d, i, n) Integrated generation rate in the vertical direction of the film; (e, j, o) Exciton generation map across the vertical direction of the active layer film as a function of wavelength.
